# The Role of Cytokines in the Fibrotic Responses in Crohn’s Disease

**DOI:** 10.3389/fmed.2017.00126

**Published:** 2017-08-07

**Authors:** Renata Curciarello, Guillermo H. Docena, Thomas T. MacDonald

**Affiliations:** ^1^Centre for Immunobiology, Blizard Institute, Barts and the London School of Medicine and Dentistry, Queen Mary University of London, London, United Kingdom; ^2^Instituto de Estudios Inmunológicos y Fisiopatológicos -IIFP-CONICET-Universidad Nacional de La Plata, La Plata, Argentina

**Keywords:** Crohn’s disease, fibrosis, fibrostenosis, cytokines, strictures, extracellular matrix, chronic inflammation

## Abstract

Crohn’s disease is an idiopathic disorder of the gut thought to be caused by a combination of environmental and genetic factors in susceptible individuals. It is characterized by chronic transmural inflammation of the terminal ileum and colon, with typical transmural lesions. Complications, including fibrosis, mean that between 40 and 70% of patients require surgery in the first 10 years after diagnosis. Presently, there is no evidence that the current therapies which dampen inflammation modulate or reverse intestinal fibrosis. In this review, we focus on cytokines that may lead to fibrosis and stenosis and the contribution of experimental models for understanding and treatment of gut fibrosis.

## Introduction

The transmural nature of the chronic inflammation in Crohn’s disease (CD) often leads to fibrosis of the deeper layers of the gut, particularly the submucosa and muscle, in about 30–50% of patients (1). At diagnosis about 27% of patients already have complications such as stenosis, fistulae, or abscesses. After 10 years of disease, the rate of complications reaches 70% (2). In ulcerative colitis (UC), which is another distinct type of inflammatory bowel disease (IBD) apart from CD and traditionally thought of as having only mucosal lesions, fibrosis occurs in about 5% of patients with longstanding disease (3). The fibrotic response causes narrowing of the gut lumen, stenosis, and eventually obstruction. It is not known why in some cases the inflammation becomes penetrating (fistulae) while in others becomes fibrotic. Complications including fibrosis are the reason why between 40 and 70% of patients require surgery in the 10 years after diagnosis (4). Traditionally, strictures were dealt with by resecting the stenotic bowel. However, in cases of multiple strictures or short bowel, other surgical techniques are being used. These include endoscopic dilation with a balloon, particularly useful for ileo-cecal strictures or anastomotic strictures following a previous resection, or stricturoplasty, where a number of different procedures are used to increase the lumen of the gut at the strictured region. There is no evidence that any medical intervention can modulate or reverse intestinal fibrosis.

The bulk of the literature on the immunology of CD has used lamina propria mononuclear cells (LPMCs) isolated from inflamed mucosa. In contrast, the literature on the immune events deep in the bowel wall is sparse because the tissue cannot be sampled at colonoscopy, and it is more difficult to purify inflammatory cells. There is a more extensive literature on the factors that control matrix production compared to matrix degradation by intestinal fibroblasts; however, the relationship to *in vivo* events is unclear. Finally, it is now appreciated that stenosis in CD also involves the cells of the circular and longitudinal muscle layers, and these cells are very poorly studied (5).

In this review, we would like to focus on the cytokines that may control the excess matrix production, which leads to fibrosis and stenosis.

## Which Cytokines are Important in Intestinal Fibrosis

Production of pro-inflammatory cytokines released during chronic inflammation leads to an initial fibrosis with prominent proliferation of myofibroblasts in the submucosa compartment (6–8). When the fibrogenic process and inflammatory stimuli persist, also smooth muscle cells differentiate and proliferate causing the smooth muscle hyperplasia/hypertrophy, characteristic of strictured CD (5, 9). The difficulty is dissecting out whether it is a combination of cytokines or if there is a single cytokine that if neutralized could prevent fibrosis.

### Transforming Growth Factor Beta (TGF-β)

Transforming growth factor beta is the best characterized pro-fibrogenic agent. The major role of one of its three isoforms, TGF-β1, in the pathogenesis of CD fibrosis has been widely studied for more than 20 years. In the 90s, it was first observed that colonic mucosa of CD patients overexpressed TGF-β, and *in vitro* studies demonstrated that TGF-β1 selectively activates extracellular matrix (ECM) synthesis, mainly collagen production, by human intestinal smooth muscle cells (10, 11). TGF-β1 receptors are also overexpressed in the intestinal mucosa of CD patients (11). On ligand binding, the receptor I kinase directly phophorylates Smad2 and Smad3, which then bind to the common mediator Smad4, and the complex translocates onto the nucleus to regulate target gene transcription (12, 13). Other pathways, such as MAPK and the phosphatidilinositol-3-kinase cascade, can also be directly activated by TGF-β1 (14). The pathway is negatively regulated by the inhibitory Smad proteins Smad6 and Smad7, which act by competing with Smad2/3 for the TGF-β receptor I kinase promoting the ubiquitination of the type I receptor for degradation in the proteasome. The expression of Smad7 is lower in mucosa overlying strictures in CD patients compared to mucosa overlying non-strictured areas, and in these sites, TGF-β expression is increased, concomitantly leading to higher levels of Smad2 and 3 phosphorylation and collagen deposition, as well as increasing the production of matrix metalloproteinases (MMPs) and their inhibitors (TIMPs) (15). Moreover, the changes produced by TGF-β1 are restricted to the strictured intestine (the Montreal B2 CD phenotype), as the phenotypes B1 (non-stricturing, non-penetrating) and B3 (penetrating) show lower levels of TGF-β1 and collagen I in the affected intestine (16). In an attempt to understand these differences, a recent study from Li et al. has demonstrated using intestinal smooth muscle cells that the increased autocrine production of IL-6 results in an abnormal STAT3 phosphorylation, which regulates increased TGF-β1, collagen and connective tissue growth factor gene expression and increased cellular proliferation, exclusively in patients with the Montreal B2 phenotype (17). Overall, the overexpression of TGF-β1 observed in CD patients to compensate the impaired regulatory function of this cytokine exerts a direct pro-fibrogenic effect through its action on mucosal myofibroblasts. In this sense, even though Mongersen, an oral antisense oligonucleotide targeting Smad7 to increase Treg cells, has been effective in inducing clinical remission in approximately 60% of patients with active CD, there is no reported evidence about its therapeutic efficacy on CD fibrosis (18–20). This highlights the different roles of TGF-β1 in inflammation and fibrosis (21). We suggest that patients should be screened for the intestinal TGF-β1 level before starting the treatment.

### IL-1β

A role of IL-1β in intestinal wound healing and stricture formation has also been proposed. IL-1 is produced predominantly by myeloid cells in response to many different pro-inflammatory stimuli such as pathogens, pathogen-associated molecular patterns, and endogenous molecules such as uric acid. After activation, the cell assembles the NLRP3 inflammasome-containing caspase 1, which cleaves pro-IL-1 into active IL-1 (22, 23). IL-1β is mitogenic for human intestinal smooth muscle cells, but strongly inhibits collagen synthesis and induces collagenase and TIMP-1 production by smooth muscle cells (24–26). Thus, the collagenolytic effect of IL-1β might be important for the initial phase of the repair process favoring the migration of mesenchymal cells to the area of injury; but if its production is sustained during the chronic inflammation, the overall effect would be an impairment of intestinal repair and a compromise in the integrity of the intestinal wall (24).

### IL-6

Human colonic smooth muscle cells secrete IL-6 after pro-inflammatory stimuli (27, 28). Li et al. have reported that patients with Montreal B2 CD phenotype have increased IL-6 production by activated mesenchymal cells. IL-6 directly induces the phosphorylation of STAT3 in muscle cells from these patients, which results in TGF-β1-driven collagen I-dependent production leading to fibrosis (17).

### IL-13

IL-13 is a type 2 cytokine with pleiotropic effects. It is produced by a variety of cell types including CD4^+^ Th2 cells, type 2 innate lymphoid cells, eosinophils, mast cells, basophils, and NK T cells, and it has emerged as a key cytokine in numerous type 2-driven diseases (29, 30). IL-13 signaling has been implicated in CD intestinal fibrosis and in fibrosis in the chronically induced trinitrobenzene sulfonic acid (TNBS) colitis model (see below) in mice (31), as well as in lung and liver fibrosis (32). IL-13 and its receptor are overexpressed in areas of fibrosis in CD patients (33). This study also identified a novel population of infiltrating IL-13Rα1^+^, KIR^+^ innate lymphoid cells into fibrotic muscle and also observed that IL-13 diminishes TNF-α-induced synthesis of MMP-1 and MMP-9, but does not directly stimulate collagen synthesis. Thus, the effect of IL-13 in intestinal fibrosis and in other tissues may be indirect, related to a downregulation of ECM degradation (33) along with induction of TGF-β1 production (34). Biologic therapy with anti-IL-13 antibodies has been used in UC with different success grades, suggesting that initial promising therapeutic strategy to neutralize IL-13 may be not clinically effective (35, 36). Nevertheless, the role of IL-13 in CD is quite debatable (37).

### TNF-α

Tumor necrosis factor family members, notably TNFSF2 or TNF-α, have a well-established inflammatory role in IBD. Various antibodies against TNF-α have been used in clinical practice in IBD for the last 20 years (38, 39). Other family members such as TL1A (TNF-like protein 1 A, TNFSF15) (40), FasL (TNFSF6) (41), LIGHT (lymphotoxin-like inducible protein that competes with glycoprotein D for binding herpes virus entry mediator on T cell, TNFSF14) (42), TRAIL (TNF-related apoptosis-inducing ligand, TNFSF10) (43), and TWEAK (TNF-like weak inducer of apoptosis, TNFSF12) (44) also contribute to the pathogenesis of IBD not only by enhancing pro-inflammatory function of T cells in the intestinal mucosa but also by directly disrupting the integrity of intestinal epithelium (45, 46). TNF-α and TL1A are potent inducers of the strictures and fibrostenotic outcome in CD: TNF-α stimulates human intestinal fibroblasts to produce excessive amounts of collagen, increases expression of MMPs, and diminishes myofibroblast mobility, which may impair mucosal healing (6, 46–48). Unfortunately, therapeutic strategies to block TNF to prevent fibrostenosis in CD were only successful in animal models (49–51). It is generally considered that anti-TNF antibodies do not prevent strictures in CD, although it has been demonstrated that they can promote TIMP secretion (52, 53) and cure CD fistulae and stenosis *in vitro* (54).

### TL1A

The role of the TL1A in colonic fibrosis has also been widely studied in terms of elucidating new molecular targets. Variants in the TLA1A coding gene *TNFSF15* has been associated with IBD, and particularly CD-associated *TNFSF15* genetic variations has been reported to contribute to enhanced induction of TL1A, resulting in severe, chronic mucosal inflammation, denoting fibrostenosis susceptibility in these patients (55, 56). Constitutive TL1A expression induces intestinal stricturing disease in two chronic colitis mouse models, and patients with higher TL1A levels (in peripheral mononuclear cells determined by ELISA) showed higher rates of intestinal strictures in CD (57). In two murine models of chronic colitis, the established colonic fibrosis was reversed using an anti-TL1A or by deletion of its receptor DR3 (57). In addition, neutralizing TL1A has resulted in lower expression of connective tissue growth factor, IL31Ra, TGF-β1 and insulin-like growth factor-1, known mediators of myofibroblast proliferation, and ECM synthesis (57). TIMP-1 was also reduced by TL1A inhibition (58). While TL1A-signaling intervention has shown promising effects in reversing colonic fibrosis in mice, there are no data in man.

### Aryl Hydrocarbon Receptor (AhR)

Signaling through AhR, a receptor for environmental toxins, can induce activation and proliferation of many cell types, such as fibroblasts (59, 60). By selectively stimulating or inhibiting AhR on isolated fibroblasts from CD patients, Monteleone et al. (61) have shown that AhR negatively regulates TNF-α- or TGF-β-driven collagen production, by affecting activation of NF-κB and Smad2/3, respectively. Moreover, they also demonstrated *in vivo* that specific stimulation of AhR in mice reduces the fibrosis associated with chronic long-term administration of TNBS in mice.

### IL-17

Since the discovery of the helper T cell subset 17 (Th17) in 2005, these cells and their characteristic cytokines (IL-17A, IL-17F. and IL-22) have been implicated in the pathogenesis of many autoimmune and inflammatory diseases, including IBD. Despite the increased production of IL-17A by mucosal T cells from IBD patients (62), the neutralizing anti-IL-17A antibody had no therapeutic effect on CD (63, 64). IL-17A signaling plays an important role in fibrogenesis of the liver (65), skin (66), and lung (67). Several studies have demonstrated that IL-17A directly interacts with colonic myofibroblasts, and it is a key triggering factor for stricture development in CD (68–70). Recently, we have shown, comparing the expression of IL-17A and its receptor in CD strictured and non-strictured gut, that IL-17A is overexpressed in strictures (71). Subepithelial myofibroblasts (SEMFs) isolated from the mucosa of CD patients express IL-17A receptors, and the IL-17A-specific response of these cells consisted on a defective migration and intensive production of collagen and TIMP-1 (71). These findings led us suggest a pro-fibrotic role for IL-17A in CD. This was further supported by the observation that the interaction between IL-17A and heat shock protein 47 (HSP47), a collagen-specific molecular chaperone involved in fibrotic disease, contributes to intestinal fibrosis in CD (72). Moreover, the expression of HSP47 was significantly elevated in intestinal tissue from patients with active CD. HSP47 and collagen I expression in isolated intestinal SEMFs were increased in response to IL-17A. Furthermore, TL1A has been identified as a local inducer of IL-17A expression in the colonic mucosa of CD patients (73), suggesting another potential mechanism that links inflammation and fibrosis in CD.

### IL-33

IL-33, an alarmin belonging to the IL-1 cytokines family, and its receptor ST2, have been implicated in the pathogenesis of IBD. Elevated expression of both molecules has been reported in inflamed mucosa from UC patients and, to a lesser extent, in CD mucosa (74, 75). Intestinal epithelial cells and SEMFs are the principal source of IL-33 in UC, but *ex vivo* studies on isolated intestinal mucosal cell populations and immunolocalization on full-thickness intestinal tissues show that IL-33 is also expressed by smooth muscle cells, endothelial cells, and adipocytes (76, 77). Several studies in acute and chronic colitis mouse models have suggested a pro-inflammatory role for IL-33 (78, 79). Interestingly, although fibrosis is usually associated with CD, it has been reported by Kobori et al. (80) and later confirmed (77) that IL-33 is expressed in activated SEMFs situated below ulcerative lesions in UC but not in CD, supporting a functional role for IL-33 in ulceration and wound healing in UC. However, it has been recently described in pediatric patients with CD that IL-33 is increased in stricturing ileitis (81). The epithelial IL-33 secretion induces recruitment and activation of eosinophils, which secrete peroxidase and IL-13, perpetuating the chronic inflammation status and priming fibroblasts to produce fibrogenic molecules (81). In these sense, IL-33 has got an indirect pro-fibrotic role, which has been described in lungs (82), skin (83), and gut (77, 84) with cell activation and increased production of TGF-β and collagen.

## Cytokine-Driven Epithelial–Mesenchymal Transition

It has been suggested that myofibroblasts can be derived from various cell sources, such as resident tissue fibroblasts (85), bone-marrow-derived CD34^+^ fibrocytes (86), or intestinal epithelial cells, in areas of fibrosis. Epithelial cells undergo morphologic and phenotypic changes in a process known as epithelial to mesenchymal transition (EMT) (87, 88). It has been recently reported that another source of activated myofibroblasts in fibrotic sites are endothelial cells that go through a phenotypic change to mesenchymal cells by endothelial to mesenchymal transition (EndoMT) process. Briefly, this process takes place when endothelial cells become delaminated and detach from the endothelial layer. Cells show the ability to change their shape from round to elongated and fusiform type, along with loss of their specific molecular markers, such as CD31/PECAM-1, von Willebrand Factor, and VE-cadherin, and novel expression of mesenchymal molecules such as α-SMA, vimentin, and type I collagen (89). Rieder et al. (90) demonstrated that EndoMT occurs in microvessels of IBD mucosa and in a mouse model of colonic fibrosis. They observed that IL-1β along with TGF-β1 and TNF-α or activated LPMCs supernatants induced morphological and phenotypic changes in human intestinal microvascular endothelial cells, as they lose their endothelial phenotype and function (LDL-uptake, migratory capacity) and they acquire *de novo* collagen synthesis capacity. Authors also showed that these cytokines induced a transient gene-specific pattern of histone modifications on activated collagen type I gene during EndoMT, supporting the hypothesis that epigenetic factors regulate fibrotic gene transcription (91). Particularly in fibrotic CD, the occurrence of epithelial to mesenchimal transition has been recently studied on patient tissue by Scharl et al. (92). In this study, several EMT markers were detected to be differentially expressed in fibrotic areas of CD mucosa, compared to non-fibrotic or non-IBD mucosa. An increased frequency of CD68^+^ cells (monocytes/macrophages) around fibrotic areas of colonic tissue also indicated a link between inflammation and fibrosis. Authors found an upregulated production of TGF-β and loss of cell membrane β-catenin expression with an increased nuclear localization in CD cells in fibrotic areas, suggesting that during EMT β-catenin translocates from the cell membrane to cytosol, and then onto the nucleus to initiate the expression of EMT-associated genes (α-SMA, vimentin, or TGF-β). Notwithstanding, it is likely that the transition from epithelial cells into myofibroblasts may not need to proceed through completely, as there is strong experimental evidence from several EMT studies demonstrating that partial EMT transition without a complete progression of epithelial cells into myofibroblasts may participate in pathologic fibrogenesis (93). However, similar studies have not been performed in EndoMT yet.

Finally, the contribution of monocytes and macrophages to fibrosis development has been recently elucidated. A novel atypical monocyte progenitor has been identified in the incitement of experimental fibrosis (94). These monocytes have a bi-lobed segmented nuclear shape and many cytosolic granules. The cellular morphology and hybrid features between monocytes and granulocytes let them be named as segregated-nucleus-containing atypical monocytes (“SatM”). Although they showed to induce fibrosis in mice, SatM did not express TGF-β. However, SatM produced large amounts of TNF-α, so the authors proposed that SatM constitutes an additional cell that may be involved in the activation of fibroblasts and initiation of fibrosis. Despite the description of these interesting findings have only been reported in lung fibrosis, it is relevant to other fibrotic disorders, such as CD fibrosis. As far as disorder-specific monocyte/macrophage cell subtypes become identified and characterized in this inflammatory disorder, they are potential targets for novel therapies for CD fibrosis with fewer side effects.

## Lessons from Murine Models of Fibrosis for New Therapeutic Strategies

Murine models of fibrosis have been helpful in studying potential therapeutic strategies for fibrosis. For example, *nintedanib* is a receptor tyrosine kinase inhibitor used in the United States for idiopathic pulmonary fibrosis, which mainly interferes with fibroblast growth factor receptor, platelet-derived growth factor receptor, and vascular endothelial growth factor receptor; these targets were identified in the bleomycin-induced lung fibrosis in mice (95).

Experimental models for intestinal fibrosis have been less productive. One of the most widely used models of Crohn’s fibrosis involves the repeated instillation (8× at weekly intervals) of TNBS into the mouse colon (47). It was demonstrated that the intestinal-induced inflammation was initiated by a Th1-mediated immune response, followed by Th17 and finally completed by IL-13-derived Th2 T cells (96). The prophylactic or therapeutic blockade of the NF-κB pathway or IL-13R alpha 2 ameliorated the fibrosis. The pro-fibrogenic role of IL-13 and TGF-β1 in colon was also described with this TNBS model (31). Therefore, the TNBS-induced fibrosis mouse model may constitute a useful biologic tool to optimize different therapies focused on suppression of fibrosis. It has been used to test the effect of vitamin D (97), anti-melanin-concentrating hormone (98), a vaccine against TGF-β (49), and novel small molecules (99) that were proposed as promising candidates for intestinal fibrosis.

The dextran sodium sulfate (DSS)-induced colitis is also widely used to induce fibrosis. The repeated administration of this chemical irritant shed light on the role of peroxisome proliferator-activated receptor gamma (PPAR-γ) on fibrosis. It was demonstrated that the induction of PPAR-γ ameliorated hepatic, lung, or intestinal fibrosis (100). Pirfenidone, an orally active small molecule comprising a modified phenyl pyridine, was used in a clinical trial for lung fibrosis, and it was showed that fibrosis can be controlled (101); a similar effect was observed with sesame oil (102) and irsogladine maleate (103) in the DSS-driven fibrosis model.

A new model of fibrosis involving subcutaneously transplanted small bowel has also been shown to be ameliorated with the administration of pirfenidone (104). Authors have demonstrated that fibrosis was inhibited through downregulation of TGF-β1 and TGF-β1-dependent intracellular cell signaling that involves Smad2/3 and MAPK. Fibroblasts proliferation and collagen production were reversed (101, 104).

A major drawback of the chemically induced models of fibrosis is that the increased collagen production is confined mainly to the submucosa, and there is no involvement of the muscle layers. This contrasts markedly with CD where there is massive involvement of the deeper layers of the gut. An exception to this is the granulomatous colitis seen when peptidoglycan polysaccharide from streptococcal cell walls are injected directly into the bowel wall of the ileum and cecum in rats (105). In this model, anti-TNF-α antibodies (50) and the natural phenol from berries, resveratrol, reduce fibrosis (106).

## Conclusion

**Figure 1:**
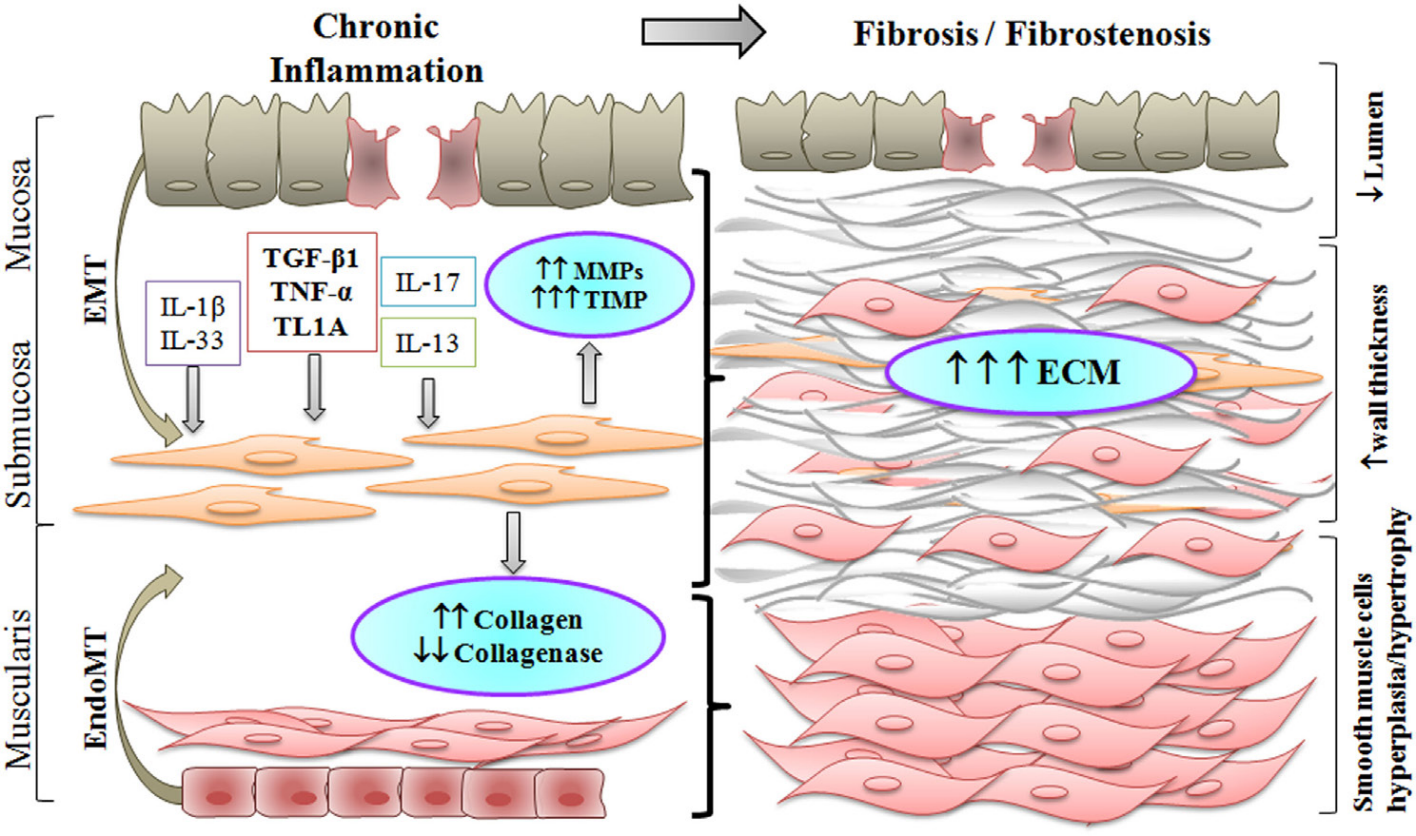
Chronic inflammation in Crohn’s disease results in fibrostenosis of the intestinal wall. Increased pro-inflammatory and pro-fibrotic stimuli activate submucosal myofibroblasts which differentiate into smooth muscle cells. Activated myofibroblasts, and fibroblasts differentiated through the epithelial to mesenchymal transition (EMT) or the endothelial to mesenchymal transition (EndoMT), produce excess of extracellular matrix (ECM). As a result, smooth muscle cell hyperplasia and hypertrophy take place, along with increased deposition of ECM, thus producing an overall thickening of the intestinal wall with narrowing of the lumen in the fibrostenotic state.

Figure 1 and Table 1 summarize the process that leads to fibrostenosis of the gut in CD. Several cytokines that are increased in the inflamed gut mucosa of CD patients can also induce fibrosis and fibrostenosis in advanced stages of the disease. We have reviewed here the most relevant cytokines that are involved directly in CD-associated fibrosis. Although some of these can directly induce fibrosis through their effect on fibroblasts activation/proliferation (TGF-β, IL-1β, TNF-α, and IL-17A), others exert an indirect effect on fibroblasts through interaction with other cell types with induction of pro-fibrotic cytokines and ECM synthesis (IL-13, IL-6, and IL-33). Compared to the huge advances in new therapies for inflammation in IBD (anti-TNFs, anti-integrins, antisense to smad7, and kinase inhibitors), there has been no progress on therapeutic modalities that might prevent or reverse fibrosis in CD patients. Many relevant studies have shed light on pathogenesis of the fibrotic process in CD. However, while *in vitro* models of intestinal fibrosis cannot model the complex intestinal architecture, *in vivo* rodent models do not fully recapitulate human disease and have really given no insight into mechanisms with translational potential. Much of the problem is that it is extremely difficult to measure fibrosis in the gut. There are many publications on imaging modalities (which we did not have enough space to cover), but these have not been used as biomarkers of antifibrotic therapies. Paradoxically, the major advances in treating bowel strictures in CD patients have been surgical strategies such as stricturoplasty.

**Table 1:** Role of the principal cytokines and molecules involved in Crohn’s disease (CD) fibrosis.

Molecules	Role in CD fibrosis
Transforming growth factor beta (TGF-β)	Induces extracellular matrix (ECM) synthesis (collagen), matrix metalloproteinases (MMPs), and TIMP production by strictured intestinal smooth muscle cells and myofibroblasts
IL-1 β	Increases collagenase and TIMP-1 production by gut smooth muscle cells
IL-6	Induces TGF-β1-driven collagen production by smooth muscle cells
TNF-α	Stimulates excessive collagen production and expression of MMPs by intestinal fibroblasts. Diminishes myofibroblast mobility
TLA1	Induces expression of connective tissue growth factor, IL31Ra, TGF-β1 and insulin-like growth factor, important mediators of myofibroblast proliferation, and ECM synthesis
IL-17A	Overexpressed in stricture CD gut. Induces collagen and TIMP-1 production by subepithelial myofibroblasts (SEMFs). Diminishes myofibroblast mobility
IL-13	Diminishes TNF-α-induced synthesis of MMP-1 and MMP-9: downregulates ECM degradation and increases collagen deposition
IL-33	Induces cell activation, TGF-β, and collagen production by SEMFs
MMPs	Matrix metaloproteinases: degrade ECM components
TIMP	MMPs tissue Inhibitor: inhibits MMPs-driven excessive degradation of ECM

## Author Contributions

TTM and RC conceptualized the review. RC and TTM provided an initial draft of the manuscript, including figure and table. TTM, GD, and RC performed the final editions. TTM receives support from the Medical Research Council, UK and from Janssen, Topivert, VH2, and GSK; GD receives grants from Argentinean National Agency for Science and Technology (PICT 2015-1249) and National University of La Plata (UNLP 12/X695); RC was supported by a special funding for young Researchers from CONICET.

## Conflict of Interest Statement

The authors declare that the research was conducted in the absence of any commercial or financial relationships that could be construed as a potential conflict of interest.
